# The longitudinal trajectory of emotion regulation and associated neural activity in patients with bipolar disorder: A prospective fMRI study

**DOI:** 10.1111/acps.13488

**Published:** 2022-08-31

**Authors:** Hanne Lie Kjærstad, Luisa de Siqueira Rotenberg, Gitte Moos Knudsen, Maj Vinberg, Lars Vedel Kessing, Julian Macoveanu, Beny Lafer, Kamilla Woznica Miskowiak

**Affiliations:** ^1^ Copenhagen Affective Disorder research Centre (CADIC), Psychiatric Centre Copenhagen Copenhagen University Hospital Rigshospitalet Denmark; ^2^ Bipolar Disorder Program (PROMAN), Department of Psychiatry University of São Paulo Medical School São Paulo Brazil; ^3^ Neurobiology Research Unit Copenhagen University Hospital Rigshospitalet Denmark; ^4^ Department of Clinical Medicine University of Copenhagen Copenhagen Denmark; ^5^ Mental Health Center, Northern Zealand Copenhagen University Hospital – Mental Health Services CPH Copenhagen Denmark; ^6^ Department of Psychology University of Copenhagen Copenhagen Denmark

**Keywords:** bipolar disorder, emotion regulation, functional neuroimaging, longitudinal, neurobiology

## Abstract

**Objectives:**

Impaired emotion regulation is a key feature of bipolar disorder (BD) that presents during acute mood episodes and in remission. The neural correlates of voluntary emotion regulation seem to involve deficient prefrontal top‐down regulation already at BD illness onset. However, the trajectory of aberrant neuronal activity during emotion regulation in BD is unclear.

**Methods:**

We investigated neural activity during emotion regulation in response to aversive pictures from the International Affective Picture System in patients with recently diagnosed BD (*n* = 43) in full or partial remission and in healthy controls (HC) (*n* = 38) longitudinally at baseline and 16 months later.

**Results:**

Patients with BD exhibited stable hypo‐activity in the left dorsomedial prefrontal cortex (DMPFC) and right dorsolateral prefrontal cortex (DLPFC) and impaired emotion regulation compared to HC over the 16 months follow‐up time. More DLPFC hypo‐activity during emotion regulation correlated with less successful down‐regulation (*r* = 0.16, *p* = 0.045), more subsyndromal depression (*r* = −0.18, *p* = 0.02) and more functional impairment (r = −0.24, *p* = 0.002), while more DMPFC hypo‐activity correlated with less efficient emotion regulation (*r* = 0.16, *p* = 0.048). Finally, more DMPFC hypo‐activity during emotion regulation at baseline was associated with an increased likelihood of subsequent relapse during the 16 months follow‐up time (β = −2.26, 95% CI [0.01; 0.99], *p* = 0.048).

**Conclusion:**

The stable DLPFC and DMPFC hypo‐activity during emotion regulation represents a neuronal trait‐marker of persistent emotion regulation difficulties in BD. Hypo‐activity in the DMPFC may contribute to greater risk of relapse.

**Significant Outcomes**

- Patients with bipolar disorder exhibit stable, trait‐related emotion regulation difficulties associated with dorsal prefrontal hypo‐activity over a 16‐month period.
- More hypo‐activity in the dorsomedial prefrontal cortex during emotion down‐regulation of negative affect was associated with an increased likelihood of subsequent relapse of mood episodes during the 16 months follow‐up.

**Limitations**

- Analyses of neuroimaging data were restricted to participants with both baseline and follow‐up data, resulting in a relatively small sample of BD patients.
- The time interval between baseline and follow‐up fMRI scans varied between participants (mean 15.8 ± 4.8 months) but was controlled for in all statistical analyses.

## 1 INTRODUCTION

Bipolar Disorder (BD) is characterized by mood instability with recurrent depressive and (hypo)manic episodes. Although effective treatments are available,^1^, ^2^ patients experience frequent residual symptoms, functional impairment^3^, ^4^ and reduced quality of life.^5^ Accordingly, BD is stated as one of the top 20 causes of the global burden of disease and years lived with disability.^6^ Emotion dysregulation is a core feature of BD, which needs to better be explored.^7^, ^8^ Emotion regulation refers to effortful and automatic attempts to downregulate, upregulate or sustain emotions that can happen before or after the emotion is generated.^9^ Neural models of emotion regulation distinguish between implicit/unconscious and explicit/controlled emotion regulation.^10^, ^11^ As such, explicit and controlled emotion regulation in healthy individuals relies on an effective interaction between emotion‐generating limbic regions, specifically the amygdala, and prefrontal cortical (PFC) regions involved in cognitive control.^12^ To date, several neuroimaging studies have shown that voluntary emotion down‐regulation of unpleasant stimuli using adaptive emotion regulation strategies, such as cognitive reappraisal, is associated with increased top‐down control of PFC on amygdala activity.^13^, ^14^, ^15^ Such findings are opposite when it comes to neuroimaging studies that analyzed voluntary emotion regulation in individuals with BD, indicating aberrant neural activation and connectivity that persist in relatively symptom‐free periods of remission^16^, ^17^, ^18^ (see^19^, ^20^ for systematic reviews). Indeed, the baseline cross‐sectional fMRI study of a slightly smaller sample showed PFC hypo‐activity during emotion regulation in remitted recently diagnosed patients compared to HC, indicating a deficient prefrontal top‐down regulation already at BD illness onset.^21^ Taken together, these studies support the notion that the neural basis of emotion dysregulation in BD involves deficient recruitment of prefrontal resources. Emotion regulation is highly relevant to BD, given that it is likely to contribute to mood instability with increased vulnerability to manic and depressive relapse. Hence, elucidating abnormalities in this aspect of cognition and its neuronal basis may provide key treatment targets to reduce relapse rates and improve mood stability.^20^, ^22^

However, no longitudinal functional neuroimaging study has investigated emotion regulation in BD. Models of neuroprogression in BD posit that the recurrence of mood episodes promotes accentuated neurocognitive deficits and changes in brain structure and cellular function over time.^23^ Yet evidence for neuroprogression in BD comes mainly from cross‐sectional studies that link greater illness progression to poorer cognitive function.^23^, ^24^ Longitudinal studies of emotion regulation have mainly investigated *self‐reported* habitual emotion regulation strategy use as predictors of subsequent mood symptoms at follow‐up, for example, Peckham et al.,^25^ Fletcher et al.,^26^ Gilbert et al.,^27^ Alloy et al.,^28^ Johnson et al.,^29^ Stange et al.,^30^ Pavlickova et al.^31^ These have generally found that maladaptive emotion regulation strategies, such as rumination and self‐blame, predicted depressive symptoms at 6‐month follow‐up^26^ and number of depressive episodes at 3.5‐year follow‐up,^28^ whereas the use of adaptive emotion regulation strategies, such as cognitive reappraisal, predicted declines in depression at 12‐months follow‐up.^29^ Nevertheless, long‐term prospective studies of the neural basis of emotion regulation in BD are critically needed to clarify whether or not BD is marked by neuroprogression and elucidate the trajectory of aberrant neural activity during emotion regulation.

### 1.1 Aims of the study

The current longitudinal study aimed to investigate: (1) trajectory of aberrant neural activity during emotion regulation in recently diagnosed patients with BD compared to HC; (2) whether neural activity during emotion regulation can be differentiated between patients who have relapsed (BD+) compared to patients who remained stable (BD‐) between baseline and follow‐up; and (4) whether neural activity during emotion regulation at baseline predicts episode relapse at follow‐up. In accordance with the neuroprogression model, we hypothesized that patients exhibit greater PFC hypo‐activity during emotion regulation over time, which is more pronounced in BD+ relative to BD‐ patients.

## 2 METHODS AND MATERIALS

### 2.1 Study design and participants

The current study is a longitudinal investigation of recently diagnosed patients with BD and HC from the ongoing Bipolar Illness Onset (BIO) study.^32^ The study participants included 43 recently diagnosed patients with BD and 38 HC, who were re‐scanned at 16‐months follow‐up. All participants were assessed in a semi‐structured interview based on the Schedules for Clinical Assessment in Neuropsychiatry (SCAN)^33^ by MDs or MSc in psychology to ascertain diagnosis status upon inclusion in the study. The recruitment of individuals with BD was made exclusively from the Copenhagen Affective Disorder Clinic, where the diagnosis was given within 2 years prior to study enrolment. With regards to showcasing the heterogeneity of the disorder, all patients referred to the clinic between aged 18 and 60 years, after having received a BD diagnosis, were eligible and thus asked to participate in the study. Individuals were diagnosed with BD according to the SCAN interview using International Classification of Diseases (ICD‐10) criteria.^34^ For the healthy control group, age‐ and sex‐matched individuals were recruited from the University Hospital, Rigshospitalet, Blood Bank. Exclusion criteria were personal or family (first‐degree relatives) history of mental disorders or substance abuse. Lack of first‐degree familial history of psychiatric illness was ascertained by thorough questioning into participants' familial history. For all participants at both timepoints, inclusion criteria included total score ≤ 14 on both the Hamilton Depression Rating Scale (HDRS‐17)^35^ and the Young Mania Rating Scale (YMRS),^36^ and exclusion criteria were a history of severe brain injury, neurological disorder (including dementia), current severe somatic illness, and/or substance abuse disorder. Patients history of mood episodes were assessed at both baseline and follow‐up and type of episode (hypomanic, manic, depression, mixed), number and duration of episodes were registered.

The authors of this paper declare that all procedures contributing to this work comply with the ethical standards of the relevant national and institutional committees on human experimentation and with the Helsinki Declaration of 1975, as revised in 2008. The study was approved by the Committee on Health Research Ethics of the Capital region of Denmark (protocol number: H‐7‐2014‐007) and the Danish Data Protection Agency, Capital Region of Copenhagen (protocol number: RHP‐2015‐023). Informed consent was obtained for all participants prior to study participation.

## 3 MEASURES

### 3.1 Emotion regulation paradigm

The fMRI task employed in this study was a well‐established voluntary emotion regulation paradigm,^13^ involving the presentation of neutral and negative images from the International Affective Picture System (IAPS).^37^ The task comprised 24 neutral and 48 unpleasant pictures, presented in three conditions: passive view of neutral images (‘passive view neutral’, 4 images), passive view of unpleasant images (‘passive view negative’, 4 images), and a voluntary down‐regulation condition that involved only unpleasant images (‘dampen negative’, 4 images), resulting in a total task time of 12 min. Participants were instructed to view the images in the ‘passive view’ conditions and to dampen their emotions in the ‘dampen negative’ conditions. To aid ecological validity of the paradigm, no guidance was given to participants regarding which emotion regulation strategy to employ in the ‘dampen negative’ conditions, thus allowing them to utilize similar strategies they employ in their daily life. Each of the three conditions was presented randomly six times, interleaved by a 16 s fixation cross on a blank screen. Each condition included different set of pictures and started with an instruction to “view” or “dampen” (4 s), followed by the presentation of four corresponding images (4 s), and concluded with a rating of unpleasantness (4 s) on a range from 1 (not at all unpleasant) to 5 (very unpleasant) (4 s), indicated by participants by using a button box with five buttons with their right hand. We used different sets of unpleasant images in the ‘passive view negative’ and ‘dampen negative’ conditions, which were matched for valence (*p* = 0.54) and arousal (*p* = 0.56) according to the IAPS normative ratings.^37^

### 3.2 Measures of functioning and quality of life

Overall functioning was assessed using the 24‐item semi‐structured interview Functional Assessment Short Test (FAST).^38^ The FAST examines functioning in six domains (autonomy, occupational functioning, cognitive functioning, financial issues, interpersonal relationships and leisure time); of which a total score of >11 indicates functional impairment.^39^ Quality of life was assessed with the European Quality of Life 5 Domain EQ‐5D^40^; and raw scores were calculated to index scores based on Danish norms.

### 3.3 MRI acquisition protocol

All fMRI scans were acquired at the Copenhagen University Hospital, Rigshospitalet using a 3‐Tesla Siemens Prisma scanner and a 64‐channel head–neck coil. A total of 366 volumes of T2*‐weighted gradient echo spiral echo‐planer (EPI) images were acquired with a field of view (FOV) of 230 x 230 mm using a 64 x 64 grid (no of slices = 23; slice thickness = 3 mm with 25% gaps in‐between; echo time = 30 ms; flip angle = 90°; repetition time [TR] = 2 s). The blood oxygen level dependent (BOLD) images were registered to T1‐weighted structural images (FOV = 230 × 230 mm; slice thickness = 0.9 mm; TR = 1900 ms; TE = 2.58 ms; flip angle = 9°; distance factor = 50%). A standard B0 field map sequence was also acquired with the same FOV and resolution as the fMRI sequence (flip angle = 60°; TR = 400 ms and TE = 7.38 ms) and used for geometric distortions correction of the BOLD images. Good image quality was determined by visual inspection of all images.

### 3.4 Analysis of fMRI data

#### 3.4.1 Pre‐processing and first‐level analysis

Data pre‐processing and first‐level analysis were conducted using fMRI Expert Analysis Tool (FEAT) v6.0^41^ from the FMRIB Software (FSL; http://www.fmrib.ox.ac.uk/fsl). Pre‐processing comprised brain extraction, B0 field distortion correction based on field map image, linear and nonlinear registration to structural space, motion correction, spatial smoothing (Gaussian kernel full width half maximum = 5 mm), and spatial normalization to the Montreal Neurological Institute (MNI) standard space. All participants' registrations were visually controlled to ascertain a good fit. The time series in each session were high pass‐filtered (to min 0.008 Hz).

The subject (1st level) analysis general linear model (GLM) included three conditions: ‘passive view neutral’, ‘passive view negative’, and ‘dampen negative’. The events were modeled as blocks convolved with a canonical hemodynamic response function with added temporal derivative and included six standard motion parameters to account for head movement. A priori contrasts of interest was emotion regulation (‘dampen negative’ > ‘passive view negative’) and emotion reactivity (‘passive view negative’ > ‘passive view neutral’). Participants with average relative movement >0.2 mm were excluded from the analyses.

#### 3.4.2 Group‐level analyses

Group‐level analyses (second‐level) were conducted in FEAT using FLAME estimation method^42^ and included two first‐level contrasts assessing *emotion regulation* (‘dampen negative’ > ‘passive view negative’ contrast) and e*motion reactivity* (‘passive view negative’ > ‘passive view neutral’).

To investigate differential change in PFC activity during emotion regulation between BD and HC (aim 1) (i.e. group x time interaction effect), we conducted a 2x2 mixed effect ANOVA for participants with complete data‐sets consisting of both baseline and follow‐up fMRI (BD *n* = 43; HC *n* = 38). To investigate differential change in PFC activity during emotion regulation between BD+ and BD‐ (aim 2), the same analysis was repeated with baseline and follow‐up fMRI data from patients with full data‐sets (BD+ *n* = 27; BD− *n* = 14). For both analyses, we used a PFC mask for small volume correction. The mask was based on peak activations in the PFC found in healthy individuals during emotion regulation (regulate > control conditions) using IAPS images in a recent meta‐analysis.^43^ Seven 10 mm spheres were constructed in FSLeyes, covering the bilateral inferior frontal gyri/VLPFC (Brodmann area [BA] 47), bilateral middle frontal gyri/DLPFC (BA 6/8), bilateral superior frontal gyri/DMPFC/DLPFC (BA 6/9), and combined to comprise the final PFC mask. We also performed an exploratory whole‐brain analysis to investigate potential differential change in neural activity within other brain regions during emotion regulation. The same analytic approach was used for the emotion reactivity contrast for exploratory purposes. All GLM models included an additional regressor to account for difference in time between baseline and follow‐up scans.^41^ Significance level for clusters was set to *p* < 0.05, corrected for multiple comparisons using the Gaussian Random Field (GRF) theory following a cluster forming threshold of *Z* > 2.57 (uncorrected *p* < 0.005).

The main‐effect of group (i.e. BD vs. HC or BD+ vs. BD‐, across time points) were assessed in a three‐level FEAT analysis. Each subject's first‐level contrasts for the two time‐points were entered in a second‐level analysis to estimate the individual mean response (fixed‐effect analysis). The contrasts from the second level were entered in a third‐level mixed effects analyses assessing between‐subjects differences in neural activity during emotion‐regulation between (i) BD vs. HC (aim 1) and (ii) BD+ vs. BD‐ (aim 2). The analyses were performed using the PFC mask, as well as exploratory whole‐brain.

Mean percent BOLD signal change within significant clusters was extracted using the featquery tool in FSL for visual illustration of the effects. Extracted BOLD signal change from these clusters was also used for post‐hoc assessment controlling for subsyndromal symptoms using mixed models analyses with group (BD, HC) and time (baseline, follow‐up) as fixed factors, controlling for time between baseline and follow‐up scans, HDRS and YMRS total scores. Finally, we compared extracted BOLD signal change during emotion regulation in BD‐I vs. HC and BD‐II vs. HC, respectively, to explore whether results would prevail across BD type. Peak activations are reported in MNI coordinates with associated anatomical structure and Brodmann area (BA).

### 3.5 Statistical analysis of behavioural data

Emotion down‐regulation success was calculated by subtracting mean of the ratings of unpleasantness in the ‘dampen negative’ conditions from the mean of the rating of unpleasantness in the ‘passive view negative’ conditions and emotion reactivity was calculated by subtracting mean ratings of unpleasantness in the ‘passive view neutral’ conditions from the mean ratings of unpleasantness in the ‘passive view negative’ conditions. To assess differential change in emotion regulation success by group over time, a linear mixed model analysis was conducted with group (BD, HC) and time (baseline, follow‐up) as fixed factors, emotion regulation as dependent variable, controlling for time between baseline and follow‐up scans. The secondary analysis was the same as above, but additionally controlling for subsyndromal depressive and manic symptoms (i.e. scores on HDRS‐17 and YMRS).

### 3.6 Associations between neural activity, behavioural ratings, subsyndromal symptoms, functioning, quality of life, and clinical characteristics

Exploratory Pearson correlation analyses were conducted for participants with baseline and follow‐up fMRI data to explore the association between extracted BOLD signal change from regions showing significant group differences and participants' behavioural ratings during emotion regulation, subsyndromal depression and mania symptoms (HDRS and YMRS, respectively), functioning (FAST total score), and quality of life across baseline and follow‐up. In patients with BD, additional correlation analyses were conducted to explore the association between extracted BOLD signal and clinical characteristics (psychotropic medication use [antidepressant, antipsychotic, anticonvulsant, lithium], BD type and illness duration).

### 3.7 Regression analyses

To explore whether aberrant neural activity during emotion regulation at baseline is associated with episode relapse or change in functioning at follow‐up (aim 3), we employed (i) logistic regression with relapse (yes/no) as outcome variable and (ii) multiple regression analyses with change in functioning (subtracted total FAST follow‐up score from baseline score) as outcome variable, respectively. Extracted BOLD signal change from clusters in which patients showed aberrant activity during emotion regulation were entered as predictor variables. Analyses were conducted within the entire BD sample, using a larger sample of patients with baseline fMRI data available (n = 60; of which: BD+: n = 41; BD‐: n = 19), and controlled for baseline mania and depression symptoms, age, sex, BD type, and time between baseline fMRI and follow‐up episode data collection. Behavioural data, correlation and regression analyses were conducted using SPSS version 25.

## 4 RESULTS

### 4.1 Participants and baseline characteristics

The study included baseline and follow‐up fMRI data from 81 participants: 43 newly diagnosed patients with BD and 38 age‐ and sex‐matched HC (Table 1). One patient was excluded due to excessive movement (original sample *n* = 82). The time interval between baseline and follow‐up scans was 16 months (mean 15.8 ± 4.8 months). Patients and controls were well‐matched with respect to age, ethnicity, sex, and years of education at baseline (*p*‐values ≥0.87) (Table 1). Information about mood episodes between baseline and follow‐up was available for 41 patients. Of these patients, 66% (*n* = 27) underwent at least one mood episode (depressive (hypo)manic or mixed episode) during the follow‐up time, whereas 34% (*n* = 14) remained in remission (Table 2).

**TABLE 1:** Baseline and follow‐up demographic variables, symptom severity, functioning and quality of life in patients with bipolar disorder and healthy controls included in the longitudinal fMRI analyses

	Baseline				Follow‐up				Group by time interaction	
	Bipolar disorder (*n* = 43)	Healthy controls (*n* = 38)	*t*‐value/*χ* ^2^	*P*‐value	Bipolar disorder (*n* = 43)	Healthy controls (*n* = 38)	*t*‐value	*P*‐value	F‐value	*P*‐value
Sex, *n* (%) female	26 (53%)	23 (47%)	0.00	1.00						
Ethnicity, *n* (%) Northern European^a^	36 (84%)	36 (95%)	2.48	0.12						
Age	26.69 (1.14)	29.95 (1.78)	−0.12	0.90	31.12 (1.14)	31.45 (1.76)	−0.16	0.87		
Years of education	15.42 (0.49)	15.32 (0.37)	0.16	0.87	15.81 (0.43)	15.57 (0.31)	0.47	0.64	0.03	0.86
HDRS	4.64 (0.57)	0.82 (0.18)	6.43	**<0**.**001**	4.35 (0.60)	0.95 (0.21)	5.36	**<0**.**001**	0.22	0.64
YMRS	2.12 (0.43)	0.55 (0.20)	3.31	**0**.**002**	1.49 (0.34)	0.68 (0.21)	1.98	0.051	1.43	0.23
FAST, total score	13.19 (1.62)	1.03 (0.25)	7.42	**<0**.**001**	8.34 (1.20)	2.08 (0.74)	4.45	**<0**.**001**	6.78	**0**.**01**
Quality of life, EQ‐5D index score	0.88 (0.02)	0.98 (0.01)	−5.09	**<0**.**001**	0.90 (0.01)	0.97 (0.01)	−3.93	**<0**.**001**	0.88	0.35

Abbreviations: EQ‐5D, European Quality of Life 5‐Dimensions; FAST, Functioning Assessment Short Test; HDRS, Hamilton Depression Rating Scale; YMRS, Young Mania Rating Scale.

^a^ Northern European ethnicity was determined by both biological parents being of Northern European decent.

**TABLE 2:** Baseline and follow‐up demographic and clinical variables and episode data in patients with bipolar disorder who relapsed (BD+) and who remained in remission (BD‐) included in the longitudinal fMRI analyses

	Baseline				Follow‐up				Group by time interaction	
	BD+ (*n* = 27)	BD‐ (*n* = 14)	*t*‐value/*χ* ^2^	*p*‐value	BD+ (*n* = 27)	BD‐ (*n* = 14)	*t*‐value/*χ* ^2^	*p*‐value	*F*‐value	*p*‐value
*Demographic and clinical variables*										
Sex, *n* (% female)	16 (59%)	8 (57%)	0.02	0.90						
Age	29.19 (8.32)	30.50 (6.15)	−0.52	0.61	30.74 (8.43)	31.79 (6.07)	−0.41	0.68	0.007	0.93
Years of education	15.27 (3.27)	15.75 (3.18)	−0.45	0.66	15.70 (3.06)	16.07 (2.50)	−0.39	0.70	0.006	0.94
HDRS	5.31 (3.74)	3.00 (3.26)	1.94	0.06	5.52 (3.92)	1.57 (2.44)	3.96	**<0**.**001**	0.98	0.33
YMRS	2.69 (3.20)	1.21 (1.67)	1.92	0.06	1.78 (2.46)	1.07 (1.73)	1.07	0.29	0.44	0.51
FAST, total score	15.00 (10.50)	8.07 (9.41)	2.07	**0**.**045**	11.00 (7.86)	3.21 (3.81)	3.48	**0**.**001**	0.05	0.83
EQ‐5D, index score	0.85 (0.10)	0.96 (0.08)	−3.51	**0**.**001**	0.88 (0.09)	0.95 (0.07)	−2.5	**0**.**02**	0.87	0.35
Bipolar type, *n* (% type II)	21 (78%)	10 (71%)	0.2	0.65						
Delay in diagnosis, years	8.04 (8.46)	4.50 (6.06)	1.39	0.17						
Illness duration, years	9.04 (8.46)	6.00 (6.23)	1.18	0.25	10.63 (8.67)	7.14 (6.42)	1.33	0.19		
Antidepressants, *n* (% yes)	6 (22%)	2 (14%)	0.37	0.54	2 (7%)	1 (7%)	0.001	0.98		
Antipsychotics, *n* (% yes)	7 (26%)	2 (14%)	0.73	0.39	8 (30%)	4 (29%)	0.005	0.94		
Anticonvulsants, *n* (% yes)	11 (41%)	6 (43%)	0.02	0.90	23 (85%)	12 (86%)	0.002	0.96		
Lithium, *n* (% yes)	12 (44%)	9 (64%)	1.45	0.23	10 (37%)	2 (14%)	2.31	0.13		
*Mood episodes between baseline and follow‐up*										
Mean follow‐up period, months	15.74 (5.00)	13.79 (3.91)	1.27	0.21						
Depressive episodes, no.	1.59 (1.31)	–								
Depressive episodes, days	104.37 (89.78)	–								
(Hypo)manic episodes, no.	0.78 (1.28)	–								
(Hypo)manic episodes, days	14.35 (36.34)	–								

Abbreviations: EQ‐5D, European Quality of Life 5‐Dimensions; FAST, Functioning Assessment Short Test; HDRS, Hamilton Depression Rating Scale; YMRS, Young Mania Rating Scale.

Baseline fMRI data were available for a larger sample of 60 patients included in the regression analyses to explore whether aberrant *baseline* activity was associated with relapse and change in functioning in newly diagnosed patients with BD (see below). Of these patients, 68% (*n* = 41) experienced at least one mood episode between baseline fMRI and the mean follow‐up period of 16 months (mean 15.9 ± 5.5 months), whereas 32% (*n* = 19) remained in euthymia.

### 4.2 Aim 1: Assessing differential change in neural activity during emotion regulation between patients and controls

#### 4.2.1 Functional magnetic resonance results

**FIGURE 1:**
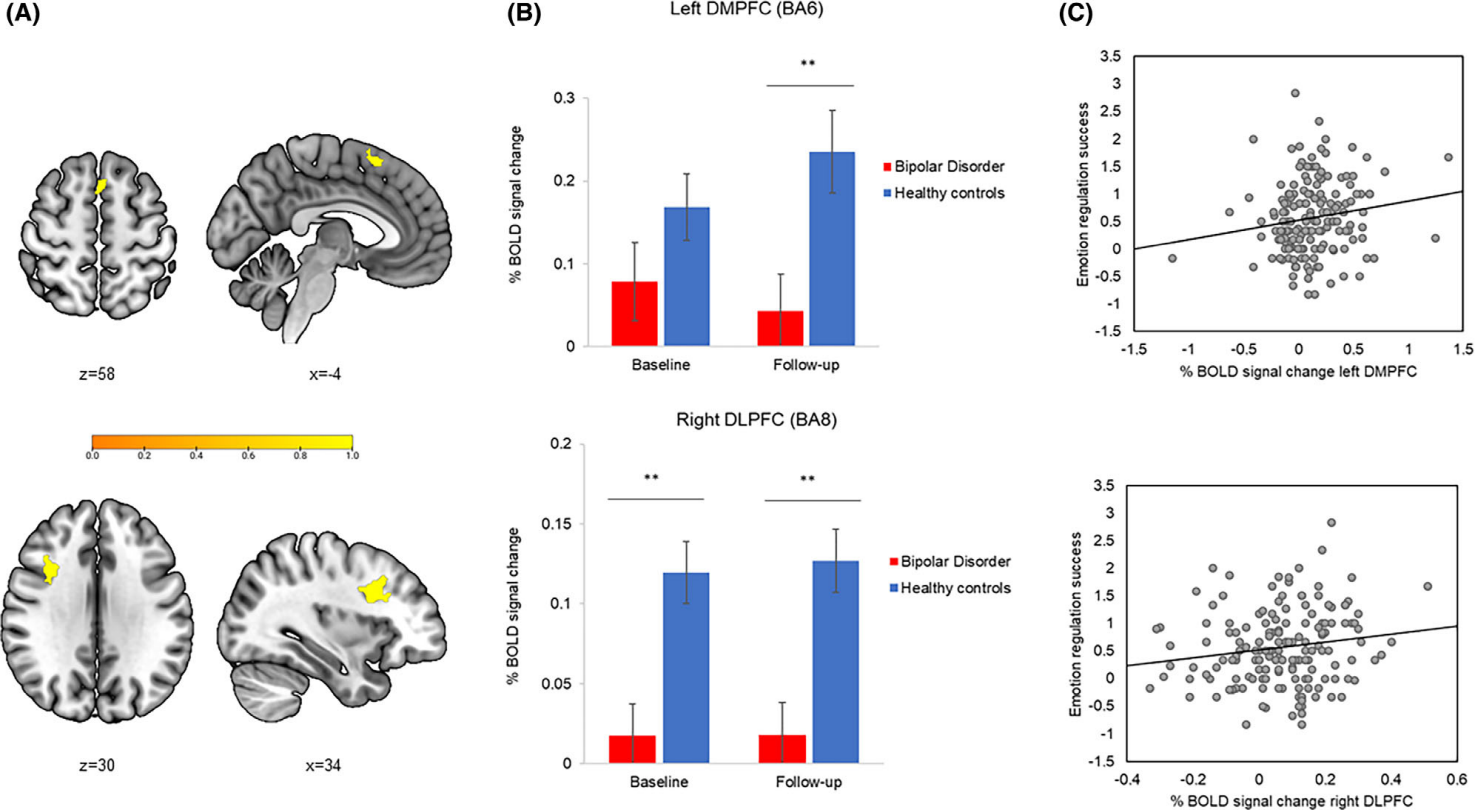
(A) Decreased activity in left dorsomedial prefrontal cortex (DMPFC; top) and right dorsolateral prefrontal cortex (DLPFC; bottom) during emotion down‐regulation (dampen negative > passive view negative contrast) in patients with bipolar disorder (*n* = 43) compared to healthy controls (*n* = 38), (B) which persist over a 16‐month follow‐up. (C) Mean percent BOLD signal change in the left DMPFC and right DLPFC during emotion regulation significantly correlated with less successful down‐regulation of emotional responses to aversive images at the behavioural level (*p*s ≤ 0.048). Error bars represent standard error of the mean. **p < 0.01

PFC analyses: We identified no significant trajectory difference (group‐by‐time interaction) during emotion regulation between patients with BD and HC. Three‐way analyses revealed a main effect of group difference during emotion regulation (‘dampen negative’ > ‘passive view negative’) in a DMPFC (BA6) cluster in the left superior frontal gyrus, driven by patients with BD generally having significantly lower activation compared to HC (Table 3; Figure 1). Post‐hoc analyses revealed that group difference remained significant after adjusting for symptom severity (*p* = 0.004).

**TABLE 3:** Main effect of group on brain activation during emotion regulation over time in patients with bipolar disorder (BD) (n = 43) and healthy controls (HC) (*n* = 38)

Search area	Region	BA	MNI			Voxels	Peak p‐value
			x	y	z		
*Main effect of group HC > BD*							
PFC ROI	Left DMPFC	6	−2	14	60	84	0.03
Whole‐brain	Right DLPFC	8	32	16	28	260	0.04

Whole‐brain analyses: We found no significant group‐by‐time interaction during emotion regulation. The whole‐brain three‐way analysis revealed a significant main‐effect of group during emotion regulation in a cluster in the right DLPFC (BA8), driven by patients with BD generally having significantly lower activation over time compared to HC (Table 3; Figure 1).

Post‐hoc analyses revealed that the group difference remained significant after adjusting for symptom severity (p < 0.001). Significant group differences between BD and HC also prevailed after limiting the BD sample to patients with BD‐I versus HC (left DMPFC: F(1, 47.04) = 4.98, *p* = 0.03; right DLPFC: F(1, 47.00) = 10.49, *p* = 0.002) and patients with BD‐II versus HC (left DMPFC: F(1,66.00) = 6.73, *p* = 0.01; right DLPFC: F(1, 65.85) = 16.35, *p* < 0.001).

#### 4.2.2 Behavioural ratings of in‐scanner emotion regulation

**FIGURE 2:**
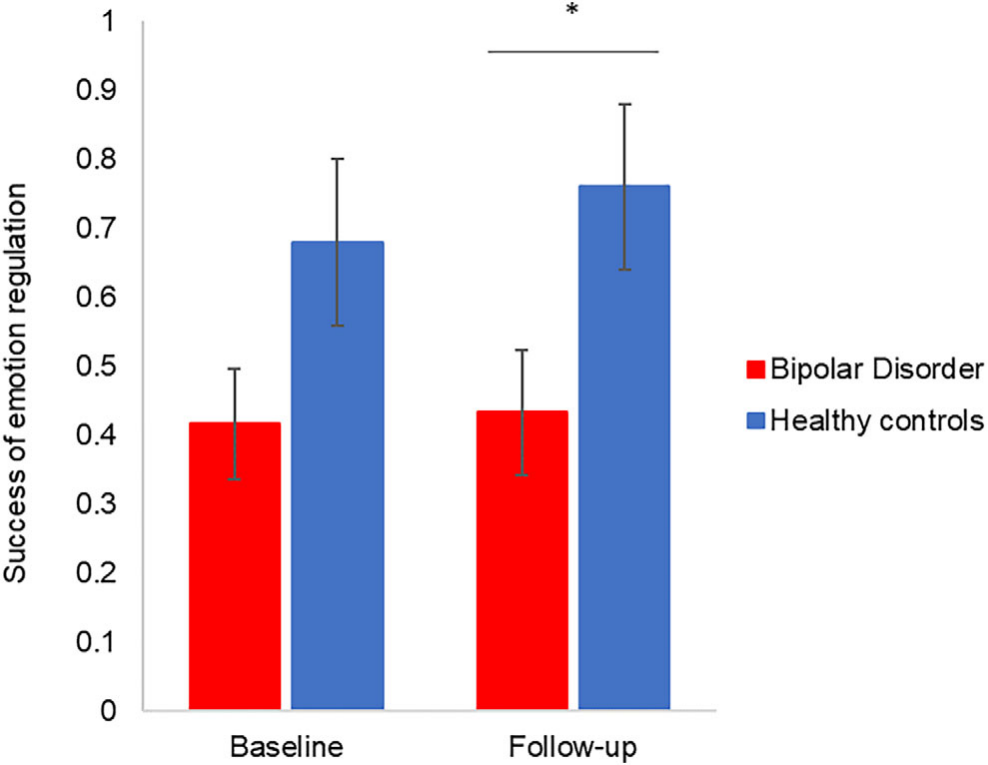
Success of emotion regulation (i.e. emotion ratings during ‘dampen negative’ conditions subtracted from emotion ratings during ‘passive view negative’ conditions) in the scanner at baseline and 16‐months follow‐up. Higher values reflect greater reduction of emotional intensity. Error bars represent standard error of the mean. *p < 0.05

For emotion regulation during fMRI, we found a significant effect of group (F[1155.99] = 7.87, *p* = 0.006): patients with BD were generally less successful at down‐regulating their emotional response to aversive images compared to HC, even after adjusting for subsyndromal depression and mania symptoms (*p* = 0.03) (Figure 2). There was no significant group‐by‐time interaction (*p* = 0.76), indicating no differential change by group over time.

#### 4.2.3 Functioning and quality of life

Analyses of functioning revealed a significant group‐by‐time interaction (F[1150.60] = 6.78, *p* = 0.01). Follow‐up analyses showed that this was driven by a significant improvement in functional impairments over time in BD patients (F[1,76.13] = 5.67, *p* = 0.02), whereas HC level of functioning remained stable over time (*p* = 0.18). Analyses of quality of life revealed a significant main effect of group (F[1149.54] = 38.72, p < 0.001): patients with BD generally had a lower quality of life compared to HC. Results revealed no significant interaction (*p* = 0.35), indicating that this lower quality of life in BD vs. HC remained stable over time (Table 1).

### 4.3 Aim 2: Assessing differential change in neural activity during emotion regulation between relapsers vs. non‐relapsers

#### 4.3.1 Functional magnetic resonance results

PFC analyses: The region of interest analysis revealed no significant group‐by‐time interaction effects nor significant difference between patients who relapsed (BD+) vs. patients who remained in remission (BD‐) in the three‐way analysis.

Whole‐brain analyses: We found no significant group‐by‐time interaction effects. Main effect or significant main effect of group in the three‐way analysis.

#### 4.3.2 Behavioural ratings of in‐scanner emotion regulation

We found no significant group‐by‐time interaction nor significant difference between the two patient groups (p‐values ≥0.51) for behavioural ratings of in‐scanner emotion regulation.

### 4.4 Emotion reactivity

#### 4.4.1 Functional magnetic resonance results

Analyses of emotion reactivity (i.e. ‘passive view negative’ > ‘passive view neutral’ contrast) revealed no significant trajectory difference (group‐by‐time interaction) nor significant difference between patients with BD vs. HC, or BD+ vs. BD‐, in the PFC and whole‐brain analyses, respectively.

#### 4.4.2 Behavioural ratings of in‐scanner emotion reactivity

We found no significant group‐by‐time interaction nor significant difference between the BD vs. HC nor BD+ vs. BD‐ (p‐values ≥0.18) for behavioural ratings of emotion reactivity.

### 4.5 Associations between neural activity, behavioural ratings, subsyndromal symptoms, functioning, quality of life, and clinical characteristics

Across the sample of participants with baseline and follow‐up fMRI data, more hypo‐activity in the left DMPFC (BA6) and right DLPFC (BA8) were associated with less successful down‐regulation of emotional responses to aversive images at the behavioural level (left DMPFC: r = 0.16, *p* = 0.048; right DLPFC: *r* = 0.16, *p* = 0.045; Figure 1C). More hypo‐activity in the right DLPFC (but not DMPFC: p‐values ≥0.26) was also associated with more subsyndromal depressive symptoms (*r* = −0.18, *p* = 0.02) and more general functional impairments (*r* = −0.24, *p* = 0.002). In contrast, BOLD response in the DMPFC and DLPFC did not significantly correlate with subsyndromal mania symptoms (*p*‐values ≥0.43) or quality of life (p‐values ≥0.09). In patients with BD, antidepressant use was significantly associated with more hypo‐activity in the left DMPFC (*r* = −0.26, *p* = 0.02) and, at trend‐level, in the right DLPFC (*r* = −0.21, *p* = 0.06). In contrast, BOLD response in the left DMPFC or right DLPFC did not significantly correlate with antipsychotic, anticonvulsant or lithium use, BD type, or illness duration (p‐values ≥0.28).

### 4.6 Aim 3: Does baseline neural activity during emotion regulation predict (i) relapse at follow‐up and (ii) decline in functioning?

Logistic regression was performed to ascertain the effects of left DMPFC (BA6) and right DLPFC (BA8) hypo‐activity during emotion regulation at baseline on the likelihood of relapse across all patients with BD. The regression model including mean percent BOLD signal in the left DMPFC as predictor was statistically significant (*χ* ^2^[7] = 19.46, *p* = 0.007). The model explained 39.0% (Nagelkerke *R* ^2^) of the variance and correctly classified 79.7% of cases. The more hypo‐activity in the left DMPFC during emotion regulation at baseline was associated with an increased likelihood of relapse (*β* = −2.26, 95% CI [0.01;0.99], *p* = 0.048). The regression model with baseline activity in the right DLPFC (BA8) as predictor variable revealed a significant model (*χ* ^2^[7] = 15.59, *p* = 0.03). However, baseline DLPFC activity did not predict relapse (*p* = 0.56).

Multiple linear regression analyses to investigate whether left DMPFC (BA6) and right DLPFC (BA8) hypo‐activity during emotion regulation at baseline was associated with decline in functioning revealed non‐significant models (left DMPFC: *p* = 0.90; right DLPFC: *p* = 0.80).

## 5 DISCUSSION

This study is the first longitudinal study to investigate the neural activity during emotion regulation in response to aversive pictures in a sample of recently diagnosed BD patients compared with healthy controls at baseline and at follow‐up 16 months later. Contrary to our hypotheses and the neuroprogression model, we found that patients with BD exhibited *stable* hypo‐activity in the left DMPFC and right DLPFC and impaired emotion regulation, as indicated by more negative emotional reactivity during attempts to dampen emotions, compared to HC over the 16 months follow‐up time. Importantly, more DLPFC and DMPFC hypo‐activity was associated with less successful emotion regulation. Further, more DLPFC hypo‐activity correlated with more subsyndromal depression symptoms and more functional impairments. Finally, more DMPFC hypo‐activity during emotion regulation at baseline was associated with an increased likelihood of subsequent relapse during the 16‐month follow‐up time (β = −2.26). Notably, these associations occurred in analyses adjusted for clinical and demographic variables.

In our well‐characterized sample, the persistent hypo‐activity in DMPFC and DLPFC over time in partially or fully remitted patients with BD is consistent with prior cross‐sectional evidence for DPFC hypo‐activity during emotion regulation in BD.^44^, ^45^, ^46^, ^47^ However, notably some other studies found *increased* PFC activity during emotion regulation in BD patients.^16^, ^17^, ^48^, ^49^ This discrepancy might be due to previous studies mostly including BD‐I in their samples, whereas our sample consist of both BD type I and II, but with an overweight of BD II. Thus differences in outcomes may be explained due to the heterogeneity of BD and the possible existence of two subgroups of BD that differ in neural activity during emotion regulation.^50^ Moreover, hypo‐activity may be associated with reduced success of emotion regulation (i.e. reduced capacity), while abnormally increased activity in DPFC could reflect less efficient emotion regulation in the absence of differences in behavioural read outs (i.e. no difference in emotion regulation performance).^51^ The model proposed by Petersen and Miskowiak^51^ was created to explain both hypo‐ and hyperactivity in DPFC during working memory based on patients' performance levels and task load, but can also be apply to studies that found no differences in their clinical groups relative to controls on emotion regulation task.^16^, ^17^, ^48^, ^49^ In line with the proposed model, emotional cognitive heterogeneity could play a vital role in understanding the hyper‐ and hypoactivity in relation to behavioural performance measures of emotion regulation. Our findings support this model, since the direction of DPFC (lower activation) is commonly observed in patients with impaired performance as well as reflects poor cognitive performance.

In line with this, explicit emotion regulation has been shown to engage lateral and dorsal frontal regions – including the DLPFC and DMPFC ‐ that are important in conscious cognitive control processes.^11^ During emotion regulation, the DLPFC has been suggested to be implicated in the cognitive selection of sensory information and response.^15^, ^52^ Indeed, the DLPFC plays a critical role in various cognitive tasks that involve executive functions, including working memory and selective attention.^53^, ^54^ Recent fMRI studies have found hypo‐activity in the DLPFC in cognitively impaired patients compared to cognitively normal patients with BD.^55^, ^56^ Poorer executive function further hampers BD patients' daily functioning,^57^ which may also have implications for adherence to treatment and has been shown to contribute to poorer clinical course and illness prognosis.^58^ Specifically, deficient emotion regulation has been found to interact with poor working memory to predict more mania symptoms at 12‐month follow‐up in patients with BD.^25^ Moreover, the DMPFC is associated with mental state inference and is recruited during tasks that require the ability to project oneself outside the present moment and focus on the perspectives and feelings of other people, such as during down‐regulation of emotion using cognitive reappraisal.^17^, ^59^, ^60^ Hence, it is possible that the less recruitment of the DMPFC during emotion regulation found in patients with BD is associated with reduced ability to utilize adaptive cognitive reappraisal strategies to regulate emotions. Given the link between emotion regulation and interpersonal functioning, this may have important implications for social skills and contribute to the more social withdrawal and restricted social networks seen in BD.^61^ Taken together, these findings indicate that a failure to recruit the dorsal PFC may underlie cognitive impairments in BD and contribute to mood instability, poorer functioning, and increased vulnerability to relapse in patients with BD.

Our observation of no differences between patients who have relapsed (BD+) compared to patients who remained stable (BD‐) over the 16 months follow‐up time suggests that DPFC hypo‐activity during emotion regulation is a stable trait in BD irrespective of mood episodes. This contrasts with the neuroprogression model,^62^ according to which BD+ patients would show exacerbation of hypo‐activity over time. However, 16 months might not be sufficient to detect neural changes associated with illness progression. Our results could, nonetheless, show *some* indication towards neuroprogression, since both DMPFC hypo‐activity and behavioural ratings of emotion regulation did not significantly differ between patients and controls at baseline, but did at follow‐up (see Figures 1 and 2), indicating a possible tendency for change if our follow‐up time interval had been longer. Also, patients who had relapsed presented more functional impairments, lower quality of life, and more subsyndromal symptoms than patients who remained stable. Hence, future studies investigating neuroprogression should consider targeting a larger time gap to infer more resolute findings.

Finally, our observation that more DMPFC hypo‐activity during emotion regulation at baseline was associated with greater likelihood of subsequent relapse was noteworthy. This finding and the association between DMPFC hypo‐activity and lower emotion regulation success may indicate that an impaired ability in BD individuals to mobilize prefrontal resources in order to dampen negative emotions could be a vulnerability trait. Lastly, more hypo‐activity in the right DLPFC correlated with more general functional impairments and subsyndromal depressive symptoms, suggesting clinical importance of DLPFC recruitment during voluntary emotion regulation for patients' daily functioning and mood stability. Taken together, DMPFC and DLPFC hypo‐activity during emotion regulation may reflect a primary deficit in BD, which contributes to poorer prognosis over time and may thus reflect key neuronal targets for strategies that aim to prevent relapse and improve functioning in patients with BD. In particular, training of emotion regulation skills – that is, recruitment of prefrontal areas to efficiently down‐regulate amygdala activity in the face of stressors and negative events ‐ may be a key element in such therapeutic interventions. Real time‐fMRI neurofeedback provides the possibility to guide mental activity based on immediate feedback of the activity in the brain region to be regulated and may be combined with emotion regulation training.^63^, ^64^ This intervention posits positive outcomes in other mental health areas such as PTSD^65^ and depression.^66^, ^67^ However, this specific intervention has not been assessed in BD patients regarding PFC hypo‐activity.

Strengths of the study included the longitudinal design with neuroimaging data acquired at two time points over a 16‐month period, the well‐established fMRI emotion regulation task^68^ and the well‐defined sample of recently diagnosed patients with BD in full/partial remission and appropriately matched controls. A limitation is that the fMRI FSL analysis requires participants full dataset (i.e. both baseline and follow‐up data). We relied on a relatively small sample of BD patients with baseline and follow‐up data, resulting in a sample size of BD+ *n* = 27 versus BD— *n* = 14 with full datasets. Although power analyses indicate that the study N = 81 is adequate to test group‐by‐time interaction effects between patients and controls in the significant neural clusters, the BD sample of N = 41 may be insufficient in detecting significant group‐by‐time interaction effects between BD+ and BD‐. Thus, results for this analysis (aim 2) should be interpreted with caution. Nevertheless, our sample of patients with longitudinal fMRI data is well above the median sample sizes of cross‐sectional fMRI studies reported in Poldrack et al.^69^ (median sample size of 28.5 as of 2015). Given the cost of fMRI, studies involving repeated fMRI testing generally suffer from low power, thus decreasing the chance of detecting a true interaction effect.^70^ Hence, future meta‐analyses and big data analyses of longitudinal fMRI data are warranted to further investigate the longitudinal trajectory of emotion regulation and associated neural activity in patients with BD. Additionally, there was some variation in the intervals between baseline and follow‐up data collection. Nevertheless, this was controlled for by including time as additional regressor in FSL and as covariate in the regression analyses. Also, in order to maintain sensitivity in the longitudinal voxel‐wise statistical model, we used a cluster forming threshold for the fMRI analyses of *p* < 0.005, which is higher than the generally recommended p < 0.001. Our findings may therefore need to be replicated in larger samples of BD patients allowing improved statistical power. Given the exploratory nature of the correlation analyses, we did not control for multiple comparisons. Yet, it is worthy to note that the significant correlations between behavioural ratings of less successful emotion down‐regulation and hypo‐activity in the left DMPFC and right DLPFC, respectively, would be reduced to a trend after FDR correction for multiple comparisons using Benjamini Hockberg (adjusted *p*‐values = 0.096). Importantly, more stringent control for multiple comparisons increases sample size requirements and decreases power and, recently, Marek et al.^71^ argue that brain‐wide association studies require thousands of participants to show reproducible correlations between functional brain activity and human behaviour. Furthermore, despite patients being diagnosed with BD within 2 years prior to study inclusion, it should be noted that patients had a substantial mean illness duration, years of untreated illness, and number of prior mood episodes. This reflects the difficulty in recruiting patients early in the course of illness due to the diagnostic delay of BD, which tends to be between five to 10 years.^72^ The heterogeneity of the sample may have introduced some confounds that could have influenced the findings. Nevertheless, the inclusion of a heterogeneous sample of patients (i.e. BD type I and II, late and early onset, etc.) was deliberately chosen in order to showcase the heterogeneity of the disorder and increase the likelihood of the results being generalizable to the disorder in general and not merely a subgroup of patients. Also, hypo‐activity in the left DMPFC and right DLPFC was exhibited in both patients with BD‐I and BD‐II compared to HC, respectively, and was not significantly associated with illness duration. Finally, the lack of a measure of self‐reported habitual emotion regulation strategy use renders it unclear how the emotion regulation impairments observed in patients with BD relate to regulatory deficits in daily life.

In conclusion, our findings provide new evidence for stable trait‐related emotion regulation deficits and its neuronal underpinnings in the early course of BD illness and its association with functioning and relapse risk. Aberrant DMPFC and DLPFC hypo‐activity during emotion regulation may thus be a key neuronal treatment target to improve BD prognosis and outcome. Future studies with longer follow up times and specific treatment target interventions are warranted to better understand possible neuroprogression correlates associated with impaired emotion regulation in BD, in addition to improving functionality, one of the major treatment objectives in BD.

## AUTHOR CONTRIBUTIONS

KWM, LVK and MV were principle investigators of the BIO study. KWM was responsible for the original study design and draft of protocol. HLK was responsible for participant recruitment, screening, and emotion regulation assessment, under the supervision of KWM. GM facilitated fMRI scanning and acquisition. HLK conducted fMRI analyses under supervision of JM. LDSR and HLK wrote the initial manuscript draft with BL and KWM. All authors contributed to interpretation of data. All authors have approved the final manuscript.

## FUNDING INFORMATION

The study is funded by grants from the Mental Health Services, Capital Region of Denmark, The Danish Council for Independent Research, Medical Sciences (DFF‐4183‐00570), Markedsmodningsfonden (the Market Development Fund 2015‐310), Gangstedfonden (A29594), Helsefonden (16‐B‐0063), Innovation Fund Denmark (the Innovation Fund, Denmark, 5164‐00001B), Copenhagen Center for Health Technology (CACHET), EU H2020 ITN (EU project 722561), Augustinusfonden (16‐0083).

## CONFLICT OF INTEREST

Maj Vinberg has received consultancy fees from Lundback, Janssen Cilag and Sunovionin the past 3 years. Gitte Moos Knudsen has received payment as speaker of Sage Therapeutics and as consultant for Sanos. Lars Vedel Kessing has within the preceding 3 years been a consultant for Lundbeck and Teva. Kamilla Woznica Miskowiak has received consultancy fees from Lundbeck and Janssen‐Cilag in the past 3 years. The remaining authors declare no conflicts of interest.

## Data Availability

The data that support the findings of this study are available on request from the corresponding author. The data are not publicly available due to privacy or ethical restrictions.
